# Does media sentiment affect stock prices? Evidence from China’s STAR market

**DOI:** 10.3389/fpsyg.2022.1040171

**Published:** 2022-12-01

**Authors:** Xiuliang Dong, Shiying Xu, Jianing Liu, Fu-Sheng Tsai

**Affiliations:** ^1^School of Business and Management, Jilin University, Changchun, China; ^2^Department of Business Administration, Cheng Shiu University, Kaohsiung, Taiwan

**Keywords:** the STAR market, media sentiment, stock price, IPO pricing efficiency, IPO first-day stock performance, machine learning

## Abstract

**Objective:**

This paper explores the impact of media sentiment on stock prices on the Shanghai Stock Exchange Science and Technology Innovation Board (hereinafter the STAR market) from a behavioral finance perspective.

**Methods:**

We collect Baidu News coverage of STAR-listed firms as the text, and measure text sentiment using a machine learning-based text analysis technique. We then empirically examine the impact of media sentiment on STAR market stock prices from two aspects: IPO pricing efficiency and IPO first-day stock performance.

**Results:**

(1) Media sentiment has no significant impact on IPO pricing efficiency, thus suggesting that institutional investors participating in such offerings are generally not affected by media sentiment. (2) Optimistic media sentiment has a positive impact on IPO first-day returns, which indicates that individual investors are more easily influenced by media sentiment and therefore likely to abandon their rational judgment. (3) Media sentiment had a greater impact on IPO first-day returns during the COVID-19 pandemic than those before it, which suggests that individual investors are more influenced by media sentiment during pandemics.

**Discussion:**

Our findings deepen the understanding of stock price formation on the STAR market, which provide a statistical basis for formulating policy directions and investment strategies.

## Introduction

Extensive behavioral finance studies have shown that the information environment of the market has a significant impact on asset prices (Broadstock and Zhang, 2019; Duan et al., 2021; Du et al., 2022). Media coverage, which is far more pervasive than traditional analyst reports, has become the most important channel of information dissemination in financial markets (Bushee et al., 2010). The presence of media outlets reduces the cost of information and the information asymmetries that exist between firms and investors (Dyck et al., 2008). However, in order to gain attention—or due to its own editorial preferences—the media may report using language with a sentimental orientation or either exaggerate or ignore certain points of view, which may result in investors having cognitive biases and thus influence their investment decisions and behaviors (Hermida et al., 2012). Against this backdrop, it is important to consider the impact of media sentiment when examining asset prices and investor behaviors, especially in the initial public offering (IPO) market, which is characterized by highly asymmetric information and an absence of historical data for investors to reference in their decision-making processes (Guldiken et al., 2017).

This paper analyzes the impact of media sentiment on the stock prices of firms listed on the Shanghai Stock Exchange Science and Technology Innovation Board (hereinafter the STAR market). We choose the STAR market as the research object because its institutional background and market atmosphere provide a natural platform for us to test the impact of media sentiment on a securities market. The STAR market is a new market comprised of scientific and technologically innovative firms established by the China Securities Regulatory Commission on 13 June 2019. Its registration-based IPO system is regarded as a groundbreaking achievement that has reformed China’s stock issuance system by allowing for deep investor participation (i.e., the “invisible hand”) in new issuance pricing. More than a decade has passed since it was first proposed, during which time its planned launch was disrupted by the sudden stock market crash in 2015 and the downturn in the following years; it was not until 2019 that the registration-based IPO system was able to be successfully launched. It can be said that the registration system piloted by the STAR market is a long-awaited institutional arrangement, thus it has received unprecedented media coverage since its inception. The STAR market has made a wide range of institutional innovations such as listing requirements and trading rules, the most critical of which is the adoption of a market-based pricing mechanism that delegates pricing power to major market participants, which marked a significant break from the previous direct IPO pricing method in China. Therefore, research on STAR market stock prices can reflect the role of media sentiment in pricing more accurately than those on other stock boards in China.

In this paper, we explore the impact of media sentiment on STAR market stock prices from two aspects: the impact on IPO pricing efficiency and the impact on IPO first-day stock performance. These two aspects correspond to the primary and secondary stock markets, respectively. According to the regulations of the Shanghai Stock Exchange, although both institutional investors and retail investors are allowed to participate in IPOs, only seven types of professional institutional investors are permitted to participate in the process of IPO inquiry on the STAR market: securities companies, fund management companies, trust companies, finance companies, insurance companies, qualified foreign institutional investors and private equity fund managers that satisfy certain conditions. Therefore, the research on the impact of media sentiment on IPO pricing on the STAR market essentially explores the impact of media sentiment on the decision-making psychology and investment behaviors of professional institutional investors. In contrast to the primary market, the secondary market comprises both institutional and individual investors, most of which are individual investors (Dong and Huang, 2022). Therefore, research on the impact of media sentiment on the first-day stock performance of IPOs can broadly reflect the impact of media sentiment on the decision-making psychology and investment behaviors of individual investors.

We find an interesting phenomenon by direct observation of some STAR-listed firms. Investors in both the primary and secondary markets show sustained enthusiasm for STAR-listed firms. For example, in the first year after the STAR market debuted (i.e., from 22 July 2019 to 22 July 2020), a total of 140 firms successfully completed IPOs. Excluding nine firms that did not generate profits, the average IPO price was ¥31.94, and the average price-to-earnings ratio was 71.05 times, both of which set a record for China’s stock market, with the latter being much higher than the average IPO price-to-earnings ratio of firms on the Shenzhen Stock Exchange Growth Enterprise Board (hereinafter the GEM market) during the same period (i.e., 23 times). The IPO price-to-earnings ratios of certain STAR-listed firms are even higher than those of GEM-listed firms in the secondary market, especially those listed in the first few months after the STAR market debuted. For instance, the IPO price-to-earnings ratio of Bestechnic is 355.03 times, that of Chipscreen Biosciences is 467.51 times, that of Sansure Biotech is 536.3 times, and that of Farasis Energy has reached 1,737.49 times. Furthermore, by examining the first-day performance of new shares in the secondary market, we find that stock prices of most STAR-listed firms show a large increase relative to their IPO price. For example, the average of 140 STAR-listed firms rose 163.87% in their first day of trading, among which the largest increase was Quantumctek, which jumped by 923.91% (i.e., from ¥36.18 to ¥370.45 per share). This phenomenon may be related to the positive media sentiment. Media is more inclined to express positive views catering to the needs of listed firms, which influences investors’ trading behavior and then pushes up stock prices (Shen et al., 2022). However, in theory, the impact of media sentiment on stock prices in the primary and secondary markets should be different. Institutional investors have more information channels available to them, a higher degree of specialization, a stronger ability to screen information, and a proven ability to invest more rationally, so it is intuitive that they may be less affected by media sentiment. On the contrary, individual investors generally lack professional knowledge and investment experience and have limited access to information. In addition, IPO firms disclose less public information and data. Media coverage has naturally become an important channel through which individual investors obtain information relevant to IPO firms, so individual investors may be affected by media sentiment to a significant extent. For this reason, this paper investigates whether and how media sentiment affects stock prices in both the primary and secondary markets of STAR-listed firms, and seeks to determine whether the observable phenomenon is caused by media sentiment.

Unlike previous studies, we build a stochastic frontier model to measure the asset pricing efficiency of the primary market by exclusively using data from the IPO market. The existing literature generally uses the IPO underpricing rate as an indicator to measure IPO pricing efficiency (Cook et al., 2006) under the premise that the secondary market is efficient—that is, the price in the secondary market always reflects the fundamental value of the firm. However, it has been repeatedly shown in empirical studies that the price discovery and resource allocation functions of China’s secondary stock market are not efficient. Cao and Dong (2006) find that stock prices in the primary and secondary markets of China’s A-share market are all overpriced, but the price bubble in the secondary market is bigger. The empirical studies on IPO pricing on the A-share (Liu and Shen, 2011) and GEM (Guo and Wan, 2011) markets have reached similar conclusions. Therefore, the degree of IPO underpricing as a measure of the rationality of IPO pricing cannot be applied to China’s stock market. Furthermore, although we focus on the first-day performance of new shares when exploring the impact of media sentiment on secondary market pricing, we also use their first five-day performance for a robustness test. Although there are no limits placed on share movements of STAR-listed firms on their first five trading days, trading is typically most active on the first day of trading, when the turnover rate is extremely high. Therefore, IPO first-day stock performance is a more accurate reflection of how individual investors judge the pricing of new shares.

Our study makes four key contributions to the literature. First, we use a machine learning-based text analysis technique to analyze media sentiment. At present, most scholars still obtain and analyze media sentiment data by manually interpretating financial news reports (Tetlock, 2007; Suleman, 2012), and their assessment of media sentiment ignores the assessment of neutral sentiment. However, we believe that manually interpretating method may not be robust enough to accurately detect media sentiment because human psychology is more prone to be affected by their own emotions when making judgements. Fortunately, current advancements in artificial intelligence make it possible to realize sentiment analysis through machine learning. Our study retrains SnowNLP, a Python library, to obtain a sentiment analysis model by constructing positive and negative training sets suitable for the stock market, which improves the reliability of media sentiment data. Second, the literature on the impact of media sentiment on the STAR market from a behavioral finance perspective is surprisingly limited, and our research therefore addresses this research deficiency. Moreover, by exploring the impact of media sentiment on IPO pricing efficiency and first-day stock performance, respectively, we investigate the extent to which institutional and individual investors are influenced by media sentiment. Third, this paper comprehensively examines the key factors that affect the stock prices of STAR-listed firms. We include variables that reflect firm attributes related to science and innovation in the regressions and an indicator variable—the follow-up investment ratio—that measures the effect of the STAR market’s innovative sponsor system. Considering that the base date of SSE Science and Technology Innovation Board 50 Index is later than the start date of the sample interval, we use a circulating shares-weighted method to compile a STAR market index, which further enriches the available indicators with respect to the STAR market sentiment. Finally, our study extends the literature related to the impact of the COVID-19 pandemic on financial markets. By exploring the changes in the relationship between media sentiment and IPO first-day stock performance before and after the pandemic declaration, we are better able to understand the impact of pandemics on individual investors’ psychology.

The main structure of this paper is organized as follows. The “Literature Review and Hypothesis Development” section reviews the literature and develops the research hypothesis. The “Research Design” section describes the sample selection, data sources, variable definitions and empirical models. The “Empirical Results and Analysis” section presents the research findings. The “Conclusion and Discussion” section discusses the conclusions, implications, limitations, and future research directions.

## Literature review and hypothesis development

Financial asset pricing methods are at the core of modern financial theory and have attracted the attention of a variety of academic circles (Mossin, 1966; Merton, 1973; Lakonishok et al., 1994). Traditional asset pricing theory is based on the neoclassical economic framework and takes the efficient market hypothesis as its basis. The neoclassicists believe that market participants have no cognitive bias and that all relevant information is fully reflected in stock prices, so investors have no incentive to obtain additional information (Sharpe, 1964; Fama, 1970; Roll, 1977). However, scholars have gradually begun to realize that the traditional asset pricing theory cannot explain certain anomalies that are observed in financial markets, such as the new issues puzzle and the small firm effect, thus its continued use has become controversial. Since the 1980s, the rise of behavioral finance has expanded traditional theory and gradually incorporated realistic constraint conditions and human psychological behaviors into asset pricing models. In terms of realistic constraints, there are certain costs associated with collecting and sorting information. When the benefits of obtaining information fail to cover the costs of doing so, investors will prefer “rational ignorance”—that is, only choosing easily accessible information and remaining rationally ignorant of all other information—which is a rational behavior in the presence of limited knowledge and information constraints. Therefore, information asymmetries are common in financial markets. Nevertheless, as an intermediary responsible for information transmission, the media can help investors overcome their rational ignorance.

By selecting, screening, repackaging and reporting relevant information, the media can enhance financial market transparency and thus lower acquisition costs (Zuckerman, 1999). Dyck and Zingales (2003) find that media coverage significantly reduces the costs and increases the efficiency of acquiring information, which in turn increases the number of informed investors in the market. Tetlock et al. (2008) argue that the media can capture unquantifiable fundamental information relevant to listed companies. Investors integrate the information reported by the media with relevant information provided by companies, then assess the value of the companies accordingly. However, because investors are not completely rational, their psychological tendencies should also be taken into account in terms of how it affects asset pricing. Investors are always willing to preprocess information according to their own experiences or reasoning, which often results in cognitive bias. Accordingly, when investors make decisions relating to asset pricing, they typically rely on their personal preferences, which supports the prospect theory proposed by Kahneman and Tversky (1979). In addition, investor sentiment also impacts asset pricing: optimistic investors usually set high prices and vice versa (De Bondt, 1993; Baker and Wurgler, 2006). Under the shock of unprecedented exogenous events, investor negative sentiment also impacts stock prices (Huynh et al., 2021).

Earlier studies on the impact of media coverage on asset pricing mostly focus on the amount of information reported and regard the media as a completely neutral information intermediary. Media coverage provides effective incremental information to investors and reduces information asymmetries, thereby affecting asset prices (Niederhoffer, 1971; Johnson et al., 2005; Bushee et al., 2010). However, the media cannot be completely neutral in a technological and commercialized society. Due to the existence of realistic factors such as differences in ownership concentration (Djankov et al., 2003), acceptance (Mullainathan and Shleifer, 2005) and financial temptation (Rinallo and Basuroy, 2009), the media will form a viewpoint that deviates from truth and objectivity to pursue its own interests, which accounts for a certain degree of subjectivity and creates a unique media sentiment. In financial markets, market participants are not completely rational. Under the influence of external information, investors’ cognition and preferences are more likely to change, and their irrationality may be exacerbated. In other words, the tone or sentiment of media coverage can affect investors’ judgment and, ultimately, asset pricing. Further, the relationship between media sentiment and prices will be strengthened in times of larger uncertainty concerning external information (Koch et al., 2022).

In recent years, scholars have begun to pay attention to the impact of media sentiment on asset pricing (Sadique et al., 2008; Tetlock et al., 2008; Loughran and McDonald, 2011; Yang et al., 2022). Some of these scholars choose to conduct media sentiment analysis by a dictionary-based approach. Tetlock (2007) explores the effect of media pessimism on market prices using data from the *Wall Street Journal*. He first conducts a text analysis of specific categories of words in the General Inquirer’s Harvard IV-4 psychosocial dictionary to create a pessimism index, and then uses the VAR model to establish the intertemporal connection between media pessimism and stock market fluctuations. His final analysis shows that media pessimism causes markets to decline, while extreme media sentiment leads to abnormally high trading volumes. Subsequently, Tetlock et al. (2008) continue the previous research and pay special attention to the impact of the proportion of negative words in financial coverage on a company’s profitability. The research documents that the more pessimistic the media sentiment, the worse its profitability is, which shows that the use of negative words in media coverage is not groundless and that media sentiment can capture fundamental information that is not easily quantified. Nevertheless, Loughran and McDonald (2011) point out that lexical sentiment in a financial context differs from that in other contexts. Most of the words identified as negative by the Harvard dictionary are typically not negative in a financial context, so they rescreen the negative words to more accurately measure media sentiment and note that it does in fact affect asset prices. Ferguson et al. (2015) and Bajo and Raimondo (2017) reach a similar conclusion. Other scholars try to understand the relationship between media sentiment and asset pricing by the use of machine learning techniques, among which Naïve Bayes algorithm is most frequently used. Antweiler and Frank (2004) is the first to apply Naïve Bayes algorithm for text analysis in the financial field. They use Naïve Bayes algorithm to extract textual sentiment information from internet stock message boards, and find that media sentiment is positively associated with stock returns. Further, Khedr et al. (2017) analyze financial news sentiment using Naïve Bayes algorithm, and construct a model to predict future stock prices based on news polarities. The model can achieve prediction accuracy results ranging from 72.73 to 86.21%. It is worth noting that machine-learning methods offer more reliable measures of disclosure sentiment than dictionary-based methods, which has been confirmed by Frankel et al. (2021). They compare the ability of dictionary-based and machine-learning methods to capture disclosure sentiment, and find that measures based on machine learning offer a significant improvement in explanatory power over dictionary-based measures. This is why we use machine learning techniques for sentiment analysis in this paper.

Considering the comparative youth of China’s stock market, there are only a few studies on the impact of media sentiment on asset pricing in the Chinese context. You and Wu (2012) first discuss asset pricing from the perspective of media sentiment. Drawing on “the spiral of silence” theory of communication, they believe that the media, as an opinion leader, can easily spread sentiment among investors, form a strong “opinion environment,” and ultimately affect the efficiency of asset pricing. As media sentiment fluctuates, asset prices gradually deviate from their underlying fundamental values. In a subsequent study, You and Zheng (2013) further find that the more optimistic the media sentiment is, the greater the tendency to underprice IPO shares will be. Wang and Wu (2015) conclude that compared with positive tone in media, negative tone is more persuasive in explaining IPO underpricing, and the more pessimistic the media sentiment is, the lower the IPO underpricing rate will be. Unlike other Chinese stock boards, the STAR market IPO system, which adopts a market-oriented inquiry mechanism and allows seven types of professional institutional investors to participate in setting the issue price, seeks to exploit the professional abilities of institutional investors in pricing assets. Compared with individual investors, institutional investors have more professional knowledge, richer investment experience, and more private information about IPOs (Funaoka and Nishimura, 2019) and are thus able to make a more comprehensive assessment of firms’ underlying value while not being easily influenced by media sentiment. Therefore, we propose:

***Hypotheses1:** The impact of media sentiment on the pricing efficiency of new issues on the STAR market is not significant.*

Individual investors can only obtain valuable information concerning IPO firms before their listing through third-party intermediaries such as the media. Furthermore, there is a relatively high proportion of individual investors and a large number of noise traders in the Chinese secondary stock market, most of whom make investment judgments based on sentiment instead of rational expectations (De Long et al., 1990; Chen et al., 2013). Therefore, such traders may be more vulnerable to media sentiment. Accordingly, we assert:

***Hypotheses2:** Media sentiment has a significant impact on the first-day stock performance of IPO shares on the STAR market.*

## Research design

### Sample selection and data sources

In this study, our initial sample consists of all firms that completed an IPO on the STAR market from 22 July 2019 to 30 April 2021. After excluding the observations for which there are missing values for key variables such as IPO pricing efficiency, this paper takes 238 firms as the final sample. We obtain the data from several sources. The financial and market transaction data are obtained from the WIND and CSMAR databases. Data on initial inquiries are manually collected from the announcements of the preliminary offline allotment results for the initial public offering of shares on science and technology innovation board. For data on media sentiment, we follow Zhang and Wu (2021) and crawl listed firms’ news coverage from Baidu News^1^ using Python software and process the news headlines to calculate the media sentiment score using Snow natural language processing (Snow NLP).

### Variables

#### Media sentiment

Following Zhang and Wu (2021), we calculate the media sentiment score (*SEN*) through the following process. First, using the abbreviations of the company names as keywords, we crawl coverage of sample firms from Baidu News using Python software and separately set the time windows to 10 days before the deadlines for inquiry of new shares and 10 days before the listing dates. Further, to test the robustness of the empirical results, we expand the time windows to use 20 and 30 days before the deadlines for inquiry of new shares and 20 and 30 days before the listing dates, respectively, which is a processing method similar to that used for lagging explanatory variables by one or two periods. A total of 43,557 media reports for IPO firms are obtained. Second, we analyze the sentiment of news headlines^2^ and calculate a sentiment orientation score for each title using SnowNLP. The closer the score is to zero, the more negative the media sentiment is; the closer the score is to one, the more positive the media sentiment is. Finally, as we calculate the media sentiment orientation scores for each IPO firm according to each time window, we obtain a mean for each time period, which is used as the main explanatory variable in this paper. However, it must be said that although SnowNLP provides a sentiment analysis model, it is constructed using data obtained from product reviews. Zhang (2021) points out that directly using models trained in other domains for text sentiment analysis will lead to the poor adaptability problem. Therefore, we retrain a new sentiment analysis model that is more suitable for the stock market by using SnowNLP through the following process. To begin with, by randomly selecting media coverage data obtained by crawling, manually identifying, and interpreting the sentimental orientation of the coverage, we collect 1000 positive and 1000 negative samples as positive and negative training sets. Next, SnowNLP walks through the training sets and tags each news headline. Then it predicts the category and probability of the text by calling the classification method of Bayes Classifier. And then, the model calculates the loss value between the prediction results and the real tag, and engages the proper gradient descent algorithm to recurrently update the model’s parameters until the loss value converges to a satisfying value. Finally, the sentiment analysis model for the stock market is well-trained. The new sentiment analysis model can calculate the probability of being positive for each news headline, which is the sentiment orientation score for each title.

#### Follow-up investment ratio

The mandatory follow-up investment system, an innovative new sponsor system used by the STAR market, mandates that relevant subsidiaries of the sponsoring institutions participate in the strategic allotment of new shares with a follow-up investment ratio of 2–5% and a 24-month lock-in period. The system is intended to align the interests of intermediaries with information advantages with those of individual investors without such advantages to distribute risk more evenly among investors while promoting rational IPO pricing and strengthening the responsibility of securities companies for follow-up supervision. The higher the follow-up investment ratio (*FUR*) is, the more money securities companies pay and the greater their alignment with the interests of individual investors is likely to be.

#### Disagreement of institutional investors’ quotation

To measure the disagreement of institutional investors’ quotations (*STDVEP*), we follow Diether et al. (2002) and manually collect and collate detailed quotation data from the IPO announcement and calculate the standard deviation for each. The higher the standard deviation, the bigger the divergence between quotations and disagreement among institutional investors are.

#### Initial public offering pricing efficiency

Unlike other studies that use the IPO underpricing rate to measure IPO pricing efficiency, we construct a stochastic frontier model to calculate the frontiers of optimal prices of new shares to measure IPO pricing efficiency (*EFF*). We assume that the premise of the first-day transaction price serves as the proxy of the firm’s value is that the efficient market hypothesis is valid in the secondary market. However, there is low efficiency with regard to resource allocation in China’s stock market (Luo and Ouyang, 2014), so this price may not be meaningful or represent the firm’s true value. Therefore, we choose to use the stochastic frontier model to calculate the IPO pricing efficiency, for which the required data are from the primary market and only include those provided by prospectuses and underwriters during valuation rounds. This method is comparatively more scientific and effective, and the frontier obtained is closer to the fundamental value of the firm, so we calculate the IPO pricing efficiency by calculating the degree to which the IPO price deviates from the effective frontier (Wang Z. et al., 2022).

#### Initial public offering first-day returns

Due to IPO firms’ relatively low exposure to investors, investors in the secondary market can simply obtain information through third-party intermediaries and IPO announcements. As the most influential information intermediary, the media can use its own channels to provide investors with information about publicly traded firms (Chen et al., 2013). Moreover, considering that there are no historical market quotations for new shares, investors’ decisions on the first day of trading will be more dependent on media sentiment. Therefore, we choose IPO first-day stock performance as the explained variable to examine the role of media sentiment in the secondary market. IPO first-day returns (*IR*), which describes the increase of stock price in the secondary market relative to the issue price, is the closest proxy for IPO first-day stock performance. In addition, as a robustness check, we replace the explained variable (IR) with the market-adjusted first-day returns (*AIR*) for verification.

#### Other variables

When examining the impact of media sentiment on the pricing efficiency of the primary market, we follow prior studies (Wang and Wu, 2015; Tutuncu, 2022a) and control the regression analysis to consider several aspects of underwriters and issuers, including underwriter reputation (*REP*), underwriting fee rate (*FR*), firm size (*SIZE*), auditor profile (*BIG4*), and return on equity (*ROE*). Furthermore, taking into account the unique scientific and innovative characteristics of STAR-listed firms, variables such as the compound annual growth rate of business revenue (*GROWTH*) and the R&D ratio (*R&D*) are added. When examining the effect of media sentiment on the first-day performance of the shares in the secondary market, we add the variables of investor sentiment and the secondary market sentiment on the issue date (*MARKET*). Among them, individual investor sentiment (*TURNOVER*) is measured by the turnover rate of new shares on the first day of trading, and institutional investor sentiment (*OVER*) is measured by the offline oversubscription ratio. The base date of the SSE Science and Technology Innovation Board 50 Index is 31 December 2019, which implies that it does not report market data prior to 30 December 2019. Therefore, we use the circulating shares-weighted method to compile the STAR market index, which selects the top 50 STAR-listed firms as constituents and sets the base date to 22 July 2019 and the base index to 1000. The specific calculation is as follows.


(1) $$\begin{array}{c} I\,n\,d\,e\,x\,o\,f\,t\,h\,e\,d\,a\,y=P\,r\,e\,v\,i\,o\,u\,s\,c\,l\,o\,s\,e\,i\,n\,d\,e\,x\\ \times \frac{\begin{array}{c} \Sigma (constituentstockclosingpriceoftheday\\ \times constituentstockweight) \end{array}}{\begin{array}{c} \Sigma (constituentstockclosingpriceofthepreviousday\\ \times constituentstockweight) \end{array}} \end{array}$$

Table 1 reports the specific definition of each variable used in this study.

**TABLE 1:** Definition of variables.

Variable	Symbol	Definition
Media sentiment 1	SEN1	The average media sentiment score on Baidu News within 10 days before the deadline for inquiry of the new shares
Media sentiment 2	SEN2	The average media sentiment score on Baidu News within 10 days before the listing date of the new shares
Media sentiment 3	SEN3	The average media sentiment score on Baidu News within 20 days before the deadline for inquiry of the new shares
Media sentiment 4	SEN4	The average media sentiment score on Baidu News within 20 days before the listing date of the new shares
Media sentiment 5	SEN5	The average media sentiment score on Baidu News within 30 days before the deadline for inquiry of the new shares
Media sentiment 6	SEN6	The average media sentiment score on Baidu News within 30 days before the listing date of the new shares
Follow-up investment ratio	FUR	The proportion of shares subscribed to by the alternative investment subsidiaries of the sponsoring institution
Disagreement of institutional investors’ quotation	STDVEP	The standard deviation of quotations for all institutions participating in preliminary inquiry
IPO pricing efficiency	EFF	The pricing efficiency estimated by the stochastic frontier model
First-day returns	IR	$\frac{\mathrm{first}-\mathrm{day}\,\mathrm{closing}\,\mathrm{price}-\mathrm{issue}\,\mathrm{price}\,\mathrm{of}\,\mathrm{new}\,\mathrm{shares}}{\mathrm{issue}\,\mathrm{price}\,\mathrm{of}\,\mathrm{new}\,\mathrm{shares}}\times 100\%$
Market-adjusted first-day returns	AIR	$\left(\frac{1+\mathrm{first}-\mathrm{day}\,\mathrm{returns}\,\mathrm{of}\,\mathrm{new}\,\mathrm{shares}}{1+\mathrm{first}-\mathrm{day}\,\mathrm{returns}\,\mathrm{of}\,\mathrm{the}\,\mathrm{market}}-1\right)\times 100\%$
IPO price-to-earnings ratio	PE	The IPO price-to-earnings ratio disclosed in the initial public offering prospectus
Firm size	SIZE	The natural logarithm of the issue volume of new shares
Industry price-to-earnings ratio	INPE	The industry price-to-earnings ratio disclosed in the initial public offering prospectus
Underwriting fee rate	FR	$\frac{\mathrm{Underwriting}\,\mathrm{fee}}{\mathrm{The}\,\mathrm{total}\,\mathrm{amount}\,\mathrm{of}\,\mathrm{IPO}\,\mathrm{funds}\,\mathrm{raised}}\times 100\%$
Underwriter reputation	REP	A dummy variable that takes the value of 1 for underwriters who gross in excess of 10% of the total proceeds and 0 otherwise, where the percentage is calculated as the sum of proceeds from IPOs underwritten by the underwriter divided by the total gross proceeds from 238 IPOs
Compound annual growth rate of business revenue	GROWTH	(revenu⁢es⁢of⁢the⁢periodrevenues⁢of⁢the⁢corresponding⁢period⁢of⁢ 3⁢years⁢ago3-1)×100%
R&D ratio	R&D	The⁢mean⁢value⁢of⁢(R&D⁢investment⁢of⁢the⁢firm⁢in⁢the⁢last⁢ 3⁢yearsRevenues⁢of⁢the⁢firm⁢in⁢the⁢last⁢ 3⁢years)×100%
Return on equity	ROE	The return on equity one year before listing
Debt to asset ratio	LEV	The debt to asset ratio one year before listing
Secondary market sentiment	MARKET	The STAR market index on the first trading day of the new shares
Individual investor sentiment	TURNOVER	The first-day turnover rate of the new shares
Institutional investor sentiment	OVER	The natural logarithm of the oversubscription ratio of offline investors
Auditor profile	BIG4	A dummy variable that takes the value of 1 for firms which select Big4 auditors and 0 otherwise
Industry effects	INDUSTRY	Based on the industrial classification standard released by the China Securities Regulatory Commission in 2012

### Models

Initial public offering pricing plays a pivotal role in the primary market, because the reasonableness of pricing, for which IPO pricing efficiency is the closest proxy, directly affects the efficiency of resource allocation in the stock market in general. Following Guo and Wan (2011) and Dong et al. (2020), we measure the IPO pricing efficiency in the STAR market through a reverse-engineered process and calculate the fundamental value of the IPO firm to estimate its deviation from the IPO price—that is, the IPO pricing efficiency. To do so, we construct the following specific stochastic frontier cost model:


(2) $$\begin{array}{l} lnPE_{i}={\text{α}}_{0}+{\text{α}}_{1}lnSIZE_{i}+{\text{α}}_{2}lnFR_{i}+{\text{α}}_{3}lnINFE_{i}\\ \;\;\;\;\;+{\text{α}}_{4}lnGROWTH_{i}+{\text{α}}_{5}lnR\&D_{i}+{\text{α}}_{6}lnROE_{i}\\ \;\;\;\;\;+{\text{α}}_{7}lnLEV_{i}+\mu_{i}+\varepsilon_{i} \end{array}$$

where *μ_*i*_* is the systematic error term, $\mu_{i}∼N^{+}\,\left(0,\sigma_{\mu }^{2}\right)$, *ε_*i*_* is the random error term, $\varepsilon_{i}∼N\,\left(0,\sigma_{\varepsilon }^{2}\right)$, and *μ_*i*_* and *ε_*i*_* are independent. The definitions of all other variables in this model are shown in Table 1.

Further, to test the impact of media sentiment on IPO pricing efficiency, we construct the following model:


(3) $$\begin{array}{c} E\,F\,F_{i}=\beta_{0}+\beta_{1}\,S\,E\,N_{i}+\beta_{2}\,F\,U\,R_{i}+\beta_{3}\,S\,T\,D\,V\,E\,P_{i}+\beta_{4}\,G\,R\,O\,W\,T\,H_{i}\\ \;\;\;\;+\beta_{5}\,R\&D_{i}+\beta_{6}\,R\,O\,E_{i}+\beta_{7}\,L\,E\,V_{i}+\beta_{8}\,S\,I\,Z\,E_{i}+\beta_{9}\,R\,E\,P_{i}\\ +\beta_{10}\,F\,R_{i}+\beta_{11}\,B\,I\,G\,4_{i}+\sum I\,N\,D\,U\,S\,T\,R\,Y+\varepsilon_{i} \end{array}$$

where *SEN*_*i*_ takes the values of *SEN1*_*i*_, *SEN3*_*i*_, and *SEN5*_*i*_, respectively.

To test the impact of media sentiment on the first-day returns of IPO shares in the secondary market, we construct the following model:


(4) $$\begin{array}{l} IR_{i}=\gamma_{0}+\gamma_{1}SEN_{i}+\gamma_{2}FUR_{i}+\gamma_{3}STDVEP_{i}+\gamma_{4}GROWTH_{i}\\ \;\;\;+\gamma_{5}R\&D_{i}+\gamma_{6}ROE_{i}+\gamma_{7}LEV_{i}+\gamma_{8}SIZE_{i}+\gamma_{9}REP_{i}\\ \;\;\;+\gamma_{10}FR_{i}+\gamma_{11}TURNOVER_{i}+\gamma_{12}OVER_{i}+\gamma_{13}BIG4_{i}\\ \;\;\;+\gamma_{14}MARKET_{i}+\sum INDUSTRY+\varepsilon_{i} \end{array}$$

where *SEN*_*i*_ takes the values of *SEN2*_*i*_, *SEN4*_*i*_, and *SEN6*_*i*_, respectively.

## Empirical results and analysis

### Descriptive statistics

Table 2 reports the descriptive statistics for the main variables in this paper. First, by observing the average values of *SEN1* and *SEN2*, it can be seen that the average value of *SEN2* is greater than 0.5000, thus indicating that media coverage of a company before its listing is more inclined to be skewed positively, which confirms the findings of Cook et al. (2006) and Gurun and Butler (2012). Second, the average *PE* of sample firms is 55.6596, which is significantly higher than the average *INPE* in the same industry (42.6873), and thus confirms the abnormally high pricing of new shares on the STAR market. Third, the average *GROWTH* of the sample firms over the past three years is 30.0984% and the average *R&D* is 10.2545%, both of which meet the requirements for scientific and innovative attributes of STAR-listed firms. In addition, there are great differences in the profitability of the sample firms, as the standard deviation of *ROE* is 10.0011, which shows that listing requirements on the STAR market have grown more inclusive in nature and are no longer singularly concerned with profitability. Fourth, with regard to underwriters, the average *REP* is 0.1555, which illustrates that less than 16% of listed firms choose high-reputation underwriters to issue new shares. The minimum of *FUR* is 2%, while the minimum of *FR* is only 1.2705%, which indicates that the underwriters’ follow-up investments may exceed the sponsors’ income. By including a long lock-in period for follow-up investments, underwriters are able to participate in the market risk associated with firms underwritten, so the follow-up investment system may in practice have a restrictive function. In addition, we note that the *FUR* of two sponsors reaches its maximum value (i.e., 10%) in cases of co-sponsorship. Fifth, STAR-listed firms have little demand for high-quality external audits, as the average of *BIG4* is 0.0714. Finally, the averages of *TURNOVER* and *OVER* are 74.5952 and 2212.8630, respectively, which reflects that both individual and institutional investors have high enthusiasm for new share listings on the STAR market.

**TABLE 2:** Descriptive statistics.

Variable	Obs	Mean	Std. dev	Min	Max
SEN1	238	0.4642	0.2640	0.0001	0.9999
SEN2	238	0.5550	0.1952	0.0336	0.9972
FUR	238	4.5488	1.0134	2	10
STDVEP	238	1.3716	1.8138	0.0146	15.0753
IR	238	1.5038	1.1024	–0.0215	7.0738
PE	238	55.6596	56.7558	12.8300	536.3000
SIZE	238	5387.3240	17226.6300	925	193846
INPE	238	42.6873	14.7629	12.9700	131.6900
FR	238	7.5285	2.2491	1.2705	21.0485
REP	238	0.1555	0.3631	0	1
GROWTH	238	30.0984	26.3425	0.5892	176.7950
R&D	238	10.2545	7.0945	1.8433	54.2933
ROE	238	12.0264	10.0011	1.3428	69.8319
LEV	238	22.9241	16.0611	1.9829	69.1950
TURNOVER	238	74.5952	5.7298	53.0751	98.9646
OVER	238	2212.8630	1459.4090	107.9693	5245.9480
BIG4	238	0.0714	0.2581	0	1
MARKET	238	1005.8270	141.7330	681.2400	1293.0100

### Effects of media sentiment on pricing efficiency in the primary market

Table 3 reports the regression results of IPO pricing efficiency in the STAR market. It can be seen that γ rejects the null hypothesis at the 1% level and passes the likelihood ratio test with an *χ^2^* of about 55.10, thus indicating that choosing the stochastic frontier cost model to measure IPO pricing efficiency is valid. IPO prices of sample firms are generally higher than their fundamental values, and the average IPO pricing efficiency is 64.16%. In addition, *INPE* supports the null hypothesis at the 10% level, thus suggesting that the IPO pricing of the sample firms differs significantly from that of other firms in the same industry. The regression coefficients of *GROWTH*, *R&D* and *ROE* are 0.1080, 0.2567 and –0.2641, respectively, and the corresponding concomitant probabilities are all less than 0.0100, thus illustrating that in terms of pricing, the STAR market is highly sensitive to listing firms’ science and technology capabilities instead of the profitability, which is consistent with its desired “hard technology” profile. With regard to underwriters, the higher the *FR* is, the lower the IPO price will be, because the underwriting fee rate is usually related to the degree of underwriting difficulty. Underwriters may therefore pursue a lower pricing strategy for firms with higher underwriting difficulty to reduce the risk of failing to realize the expected capital gains after listing (Jamaani and Ahmed, 2020).

**TABLE 3:** Regression results of the IPO pricing efficiency model on the STAR market.

Variable	Regression	Std. dev	t-statistics	*P*-value
	coefficient			
SIZE	–0.0216	0.0396	–0.5460	0.5856
FR	–0.1448	0.0661	–2.1897	0.0295
INPE	–0.0006	0.0754	–0.0078	0.9938
GROWTH	0.1080	0.0339	3.1812	0.0017
R&D	0.2567	0.0464	5.5326	0.0000
ROE	–0.2641	0.0470	–5.6205	0.0000
LEV	–0.0396	0.0385	–1.0290	0.3046
c	3.6162	0.5143	7.0313	0.0000
γ	0.9473	0.0212	44.7053	0.0000
LR test	χ^2^ = 55.10(0.0000)			
Mean value of EFF	64.16%			

Panel A of Table 4 shows the OLS regression results of media sentiment on IPO pricing efficiency. The effect of media sentiment on IPO pricing efficiency—that is, the performance of the primary market—is not statistically significant at conventional levels as the concomitant probability greater than 0.0100, which confirms *Hypothesis1*. This is because the investors in the primary market are typically highly experienced institutional investors who have a better grasp of how to properly value firms and are better equipped to process and verify relevant information (Neupane et al., 2016). Hence, they are less influenced by media sentiment. The variable that has the most pronounced positive impact on IPO pricing efficiency is *FUR*, as its coefficients are all significantly positive at the 5% level in the three regression models. From the evidence above, we conclude that the mandatory follow-up investment system of the STAR market has played a key role in enhancing the efficiency of IPO pricing. Through this mechanism, securities companies actively engage in strengthening their own responsibilities and gradually become the true gatekeepers of the IPO market. The arrangement by which sponsors only recommend, but do not guarantee, the quality of listed firms no longer holds. Similarly, due to the restrictive two-year lock-in period, securities companies are forced to carefully judge the quality of IPO projects to ensure that their follow-up investment projects are profitable and promote pricing efficiency in the primary market.

**TABLE 4:** Regression results of media sentiment on IPO pricing efficiency and IPO first-day returns.

Variable	(1)	(2)	(3)
**Panel A: Regression results of media sentiment on IPO pricing efficiency**			
SEN1	–0.0218 (–0.5253)		
SEN3		–0.0369 (–0.8051)	
SEN5			–0.0267 (–0.5513)
FUR	0.0334** (2.3846)	0.0332** (2.3744)	0.0333** (2.3914)
STDVEP	–0.0103 (–0.7735)	–0.0103 (–0.7759)	–0.0102 (–0.7690)
GROWTH	–0.0002 (–0.2388)	–0.0001 (–0.2175)	–0.0001 (–0.2218)
R&D	–0.0025 (–0.9673)	–0.0025 (–0.9748)	–0.0025 (–0.9784)
ROE	–0.0040** (–2.1563)	–0.0040** (–2.1657)	–0.0040** (–2.1602)
LEV	–0.0006 (–0.6432)	–0.0006 (–0.5905)	–0.0006 (–0.6263)
SIZE	–0.0109 (–0.4651)	–0.0111 (–0.4780)	–0.0114 (–0.4921)
REP	0.0060 (0.1704)	0.0060 (0.1705)	0.0050 (0.1426)
FR	–0.0004 (–0.0546)	–0.0005 (–0.0649)	–0.0004 (–0.0576)
BIG4	–0.0246 (–0.4611)	–0.0247 (–0.4619)	–0.0226 (–0.4260)
Constant	0.6147*** (2.6892)	0.6196*** (2.7319)	0.6201*** (2.7352)
INDUSTRY	Control	Control	Control
Observations	238	238	238
Adjusted R^2^	0.2960	0.2890	0.2910

**Variable**	**(I)**	**(II)**	**(III)**

**Panel B: Regression results of media sentiment on IPO first-day returns**			
SEN2	0.9806** (2.3862)		
SEN4		0.7061** (2.1054)	
SEN6			0.8880** (2.1323)
FUR	0.0786 (0.8855)	0.0765 (0.8466)	0.0746 (0.8513)
STDVEP	–0.0462 (–0.8448)	–0.0473 (–0.8408)	–0.0506 (–0.9020)
GROWTH	0.0051 (1.3942)	0.0050 (1.3403)	0.0049 (1.3386)
R&D	0.0166* (1.7500)	0.0172* (1.8272)	0.0166* (1.7543)
ROE	–0.0033 (–0.2811)	–0.0035 (–0.2924)	–0.0029 (–0.2409)
LEV	–0.0033 (–0.6687)	–0.0031 (–0.6223)	–0.0034 (–0.6765)
SIZE	0.4448** (2.4610)	0.4485** (2.4944)	0.4423** (2.4405)
REP	0.1566 (0.8438)	0.1385 (0.7530)	0.1567 (0.8373)
FR	0.1202*** (2.6941)	0.1193*** (2.6878)	0.1197*** (2.6937)
TURNOVER	0.0472*** (3.7312)	0.0471*** (3.7034)	0.0476*** (3.7469)
OVER	0.0163 (0.1907)	0.0220 (0.2563)	0.0178 (0.2075)
BIG4	0.2612 (0.9087)	0.2640 (0.9312)	0.2495 (0.8704)
MARKET	0.0029*** (5.6045)	0.0029*** (5.6568)	0.0029*** (5.6034)
Constant	–10.1328*** (–4.2554)	–10.1267*** (–4.2712)	–10.1228*** (–4.2516)
INDUSTRY	Control	Control	Control
Observations	238	238	238
Adjusted R^2^	0.2960	0.2890	0.2910

White (1980) heteroskedasticity-consistent t-statistics are in parentheses. *, ** and *** indicate significance at the 10, 5, and 1% levels, respectively.

### Effects of media sentiment on IPO first-day returns in the secondary market

Panel B of Table 4 presents the OLS regression results of media sentiment on IPO first-day returns. The regression coefficients of *SEN2*_*i*_, *SEN4*_*i*_, and *SEN6*_*i*_ are all significant at the 5% level, thus indicating that media sentiment has a significant and positive impact on the first-day returns of new shares—that is, the more positive the media sentiment is, the higher the IPO first-day returns will be. One possible explanation for this phenomenon, as suggested by Wang X. et al. (2022), is that many noise traders participate in the secondary market, especially on the first day of trading. Unlike professional institutional investors who are able to obtain private information, individual investors are at an information disadvantage and can only obtain actionable information through third-party intermediaries such as the media. Furthermore, individual investors’ relative inability to evaluate information makes them more vulnerable to media influence. These irrationality characteristics of individual investors eventually cause asset prices and to deviate from their underlying fundamental values. Chan and Fong (2004) argue that bullish media sentiment will lead to optimistic investor expectations, which will in turn inflate the prices of new shares. The findings of this paper also confirm this view. Thus, *Hypothesis2* is supported.

Panel B of Table 4 shows that the regression coefficient of *TURNOVER* rejects the null hypothesis at the 1% level, thus suggesting that the more bullish the individual investor sentiment is, the higher the IPO first-day returns will be, which is consistent with the view of Cook et al. (2006). Interestingly, the regression coefficient of *FUR* supports the null hypothesis at the 10% level, which indicates that the impact of the follow-up investment system on IPO first-day returns is not significant and illustrates, to some degree, that its expected supervisory function has not been effective. Furthermore, due to the predominance of individual investors on the first day of trading and thus the prevalence of irrational investment behaviors, disagreement among institutional investors with regard to pricing new shares cannot change individual investors’ psychological states, which are characterized by blind optimism and overconfidence. Therefore, it is reasonable that the regression coefficient of *STDVEP* is not statistically significant. The regression coefficient of *MARKET* is significantly positive with a concomitant probability of less than 0.0100, which implies that the more active the market is, the higher the IPO first-day returns will be.

### Additional analysis: Effects of the COVID-19 pandemic

Since the outbreak of the COVID-19 pandemic, a growing stream of literature has discussed the upheaval it caused in the financial markets and its impact on investors (Al-Awadhi et al., 2020; Schell et al., 2020; Bouri et al., 2021). Studies find that during the COVID-19 pandemic, individual investor participation increased (Tutuncu, 2022b), and their investing psychology became irrational and emotional (Silalahi et al., 2020), which may in turn influenced the relationship between media sentiment and stock prices in the secondary market.

To examine the impact of the pandemic on the relationship between media sentiment and IPO first-day stock performance, we take 11 March 2020 (i.e., the date on which the World Health Organization declared COVID-19 a pandemic) as the time node and separate our research sample into IPOs after the pandemic declaration (hereinafter COVID IPOs) and those before it (hereinafter non-COVID IPOs), then test the impact of media sentiment on first-day returns of COVID IPOs and non-COVID IPOs, respectively. Table 5 reports the OLS regression results. The regression coefficient of media sentiment on COVID IPO first-day return (1.0324) is greater than that on non-COVID IPO first-day return (0.7324), and both are significant at the 10% level, which indicates that IPO first-day returns after the COVID-19 pandemic declaration are more significantly impacted by media sentiment. We argue that there are two reasons behind this phenomenon. One is that irrational individual investor participation increased with institutional investor decreasing during the pandemic (Tutuncu, 2022b), but the trading behavior of individual investors became more emotional. The other is that the outbreak of the COVID-19 pandemic created great uncertainty in the stock market, which influenced investors’ psychological and behavioral factors (Hoffmann et al., 2013; Nabil and Elalfy, 2022). Greater market uncertainty causes investors to search for more information (Bonsall et al., 2020), which makes media sentiment more likely to influence individual investors and in turn affect stock prices on the secondary market.

**TABLE 5:** Regression results of media sentiment on the first-day returns of COVID IPOs and non-COVID IPOs.

Variable	(I) All	(II) Non-Covid IPOs	(III) Covid IPOs
SEN2	0.9806** (2.3862)	0.7324* (1.8170)	1.0324* (1.7517)
FUR	0.0786 (0.8855)	–0.0063 (–1.6501)	0.0050* (1.6954)
STDVEP	–0.0462 (–0.8448)	0.0312*** (2.9087)	0.0060 (0.3287)
GROWTH	0.0051 (1.3942)	0.0614*** (4.5735)	–0.0056 (–0.7970)
R&D	0.0166* (1.7500)	–0.0050 (–1.1318)	–0.0015 (–0.2287)
ROE	–0.0033 (–0.2811)	0.1798 (1.0662)	0.4863* (1.9182)
LEV	–0.0033 (–0.6687)	0.0176 (1.2190)	0.0895*** (4.3761)
SIZE	0.4448** (2.4610)	0.3296** (2.1523)	–0.6182*** (–2.8401)
REP	0.1566 (0.8438)	–0.0410 (–0.4224)	0.0814 (0.6850)
FR	0.1202*** (2.6941)	–0.0643 (–0.7034)	–0.0113 (–0.1812)
TURNOVER	0.0472*** (3.7312)	0.0944** (2.4761)	0.1320** (2.1148)
OVER	0.0163 (0.1907)	0.2526 (1.3922)	–0.0188 (–0.0634)
BIG4	0.2612 (0.9087)	0.4072 (1.2125)	0.0770 (0.1692)
MARKET	0.0029*** (5.6045)	0.0047*** (8.8569)	0.0027*** (2.7274)
Constant	–10.1328*** (–4.2554)	–9.0939*** (–3.6850)	–8.9668*** (–2.4195)
INDUSTRY	Control	Control	Control
Observations	238	85	153
Adjusted R^2^	0.2960	0.7250	0.3170

White (1980) heteroskedasticity-consistent t-statistics are in parentheses. *, ** and *** indicate significance at the 10, 5, and 1% levels, respectively.

### Robustness tests

To check for robustness, this study conducts three *post-hoc* tests. The results are presented in Table 6. First, we control for potential bias resulting from the selection of the sample period. The establishment of the STAR market attracted a great deal of investor attention, thus the market was quite lively and IPO firms became media “darlings” to a great extent. In order to report noteworthy coverage among the numerous news reports, the media will use more words that carry a sentimental orientation in its coverage and its sentiment will become more radical as a result. Therefore, we follow the robustness test method of that used in Zhang and Wu (2021) to eliminate the data of listed firms in the first three months after the STAR market debuted, then re-run the regression analysis. Columns (1), (2), (3), (I), (II) and (III) of Table 6 show the regression results after changing the sample period. There are no substantial changes from the previous tests, which confirms that the conclusions of this paper are robust. Furthermore, we control for potential bias due to the variable measurement methods. The measurement of IPO pricing efficiency is the basis of this research, so following Guo and Wan (2011), we substitute IPO price for IPO price-to-earnings ratio as the explained variable and re-fit the stochastic frontier cost model. Then, we take the new pricing efficiency obtained as the explained variable to test the impact of media sentiment on the pricing efficiency of the primary market. In addition, we replace first-day returns with market-adjusted first-day returns for further verification. Columns (4), (5), (6), (IV), (V) and (VI) of Table 6 present the regression results after replacing the explained variables, which confirm the robustness of our results. Finally, considering that the stock prices of STAR-listed firms on all of the first five trading days fully reflect individual investors’ involvement due share movements having no daily limits during this period, we further examine the effect of media sentiment on IPO first five-day returns to check the robustness of our results. We use IPO first five-day returns (*IR5*) and market-adjusted first five-day returns (*AIR5*) as proxies for stock performance in the secondary market as explained variables, respectively. The key explanatory variable is the average media sentiment score on Baidu News within five days of the listing date of the new shares (*SEN7*). Correspondingly, *TURNOVER* and *MARKET*, as control variables, are also adjusted to indicate observations that fall within five days of the listing date of the new shares. Leaving other control variables unchanged, we re-run the regression model. Panel C of Table 6 presents the regression results of media sentiment on IPO first five-day returns. It can be seen that media sentiment still has a significant impact on first five-day returns, which again confirms the results of this paper.

**TABLE 6:** Results of robustness tests.

Variable	Changing the sample period			Replacing the explained variables		
		
	(1)	(2)	(3)	(4)	(5)	(6)
**Panel A: Robustness test of the impact of media sentiment on IPO pricing efficiency**						
SEN1	–0.0133 (–0.3000)			0.0008 (0.0300)		
SEN3		–0.0259 (–0.5400)			–0.0158 (–0.4500)	
SEN5			–0.0061 (–0.1200)			–0.0212 (–0.5700)
Control variables	YES	YES	YES	YES	YES	YES
INDUSTRY	YES	YES	YES	YES	YES	YES
Observations	205	205	205	238	238	238
Adjusted R^2^	0.3097	0.3103	0.3095	0.3690	0.3696	0.3700

**Variable**	**Changing the sample period**			**Replacing the explained variables**		
		
	**(I)**	**(II)**	**(III)**	**(IV)**	**(V)**	**(VI)**

**Panel B: Robustness test of the impact of media sentiment on IPO first-day returns**						
SEN2	1.1117** (2.2400)			1.0175** (2.3794)		
SEN4		0.8303** (2.0800)			0.7488** (2.1713)	
SEN6			0.9810** (1.9600)			0.9377** (2.1680)
Control variables	YES	YES	YES	YES	YES	YES
INDUSTRY	YES	YES	YES	YES	YES	YES
Observations	205	205	205	238	238	238
Adjusted R^2^	0.4246	0.4200	0.4194	0.2950	0.2880	0.2910

**Variable**	**(i)**	**(ii)**
	**IR5**	**AIR5**

**Panel C: Robustness test of the impact of media sentiment on IPO first 5-day returns:**		
SEN7	0.6698*	0.6767*
	(1.7319)	(1.7516)
Control variables	YES	YES
INDUSTRY	YES	YES
Observations	238	238
Adjusted R^2^	0.1720	0.1720

White (1980) heteroskedasticity-consistent t-statistics are in parentheses. *, ** and *** indicate significance at the 10, 5, and 1% levels, respectively.

## Conclusion and discussion

### Conclusion

This paper analyzes 238 STAR-listed firms the perspective of behavioral finance for the period from 22 July 2019 to 30 April 2021 and systematically examines the impact of media sentiment on stock prices on the Shanghai Stock Exchange STAR market from two aspects: IPO pricing efficiency and IPO first-day stock performance, which also reflect the influence of media sentiment on institutional and individual investors. Furthermore, we investigate the impact of the COVID-19 pandemic on the relationship between media sentiment and IPO first-day stock performance on the STAR market. We obtain data for media coverage of STAR-listed firms by crawling Baidu News using Python software and measure media sentiment using a machine learning-based text analysis technique. The results suggest the following. (1) Media sentiment has no significant impact on IPO pricing efficiency on the STAR market. This implies that institutional investors that participate in the primary market are not influenced by media sentiment, which is a likely result of their analytical sophistication and investing experience. However, it remains true that IPO prices of STAR-listed firms are obviously overestimated, with an average IPO pricing efficiency of 64.16%. This phenomenon can be explained by Derrien (2005)—that is, rational institutional investors participating in the IPO process intentionally and opportunistically increase the prices of new shares after observing the hot market atmosphere. (2) The more optimistic the media sentiment is, the higher the IPO first-day returns will be, which indicates that the sentimental orientation of media reports significantly induces generally irrational individual investors to overbid for and thus drive up the prices of newly issued shares. In addition, the results remain robust after extending IPO first-day returns to IPO first five-day returns. (3) Media sentiment has a greater impact on IPO first-day returns after the COVID-19 pandemic declaration than before the declaration. This finding suggests that irrationality among individual investors intensifies during pandemics, which makes them more susceptible to media sentiment and thus strengthens the relationship between media sentiment and stock prices. The above conclusions still hold after a series of robustness tests through which we change the sample period and choose alternative variable measurements.

### Implications

This study adds to emerging research on how media sentiment affects stock prices. By taking the perspective of behavior finance, we provide a novel explanation for the mechanisms that affect stock price formation on the STAR market. Our study also has implications for policy formulation and investment behavior based on the above findings. First, it is necessary to strengthen the regulations and guidance on media behaviors and encourage the media to play an active role in financial markets. Our empirical evidence suggests that media sentiment has a great impact on the judgment of individual investors, and we believe that such influence will increase along with the increasingly high development of information technology. Therefore, regulators should incentivize the media to report with objectivity and neutrality while punishing non-compliance and overt or self-interested biases. Second, the “gatekeeper” function of sponsors and lead underwriters should be fortified and the follow-up supervision responsibility of securities companies should be strengthened. Our results demonstrate that the mandatory sponsor follow-up investment system has improved the efficiency of stock pricing, but not the effectiveness of follow-up supervision. Hence, regulators should clarify the obligations of securities companies and provide institutional guarantees to ensure that they continue to safeguard the market after the IPO process is complete. Third, public health crisis events should be paid attention to, as they might induce market volatility as well as market disruption. We find that the COVID-19 pandemic declaration reinforced the positive impact of media sentiment on IPO first-day returns. Therefore, regulators need to reduce the market uncertainty caused by the pandemic by ensuring the authenticity and timeliness of information disclosure of listed firms, which in turn mitigates the adverse impact of media sentiment on the stock market during the pandemic. Finally, investors need to make value investment. The data show that most STAR stocks have high first-day returns in the secondary market, but our research has confirmed that STAR stock prices are already overvalued in the primary market, which indicates that the stock price bubble exists and investment risk is high. However, compared with mature capital markets, there is indeed a more speculative atmosphere characterized by irrational behavior in China’s stock market, which is also confirmed by our result that media sentiment affecting stock pricing in the secondary market is a function of the irrational behaviors of individual investors. Therefore, investors should be toward value investing and away from speculative short-term trading, especially during pandemics.

### Limitations and future research

This paper is subject to certain limitations that may present avenues for further research. Zhang et al. (2022) find that in China’s bond market, investors’ reactions to good and bad news are asymmetric. In addition, the asymmetric effect of sentiment has been confirmed to exist in the stock market (Akhtar et al., 2013). Hence, we believe that positive and negative media sentiment should have different effects on STAR market stock prices. However, due to the relative youth of the STAR market, our research sample is small. If we were to classify the existing research samples by media sentiment to re-test our hypotheses, we would not be able to ensure the validity of the empirical results due to the small sample size. Therefore, we did not carry out this step and will continue to deepen our research when a larger sample is available. In addition, as we are limited by time and manpower, we did not further analyze the mechanism of impact of media sentiment on stock prices and plan to do so in future research efforts.

## Data availability statement

The raw data supporting the conclusions of this article will be made available by the authors, without undue reservation.

## Author contributions

XD conceived and supervised the study. SX conducted literature review and wrote the manuscript. JL contributed to the acquisition, analysis, and interpretation of data for the work. F-ST contributed to construct the model and write the manuscript. All authors contributed to the article and approved the submitted version.

## Footnotes

1 We choose Baidu News as the platform to observe the media coverage of IPO firms for three reasons. First, as the largest Chinese search engine, Baidu has more stable servers and the advantage of being a Chinese information search engine, so it ensures that data crawling is technically feasible. In addition, the news sources used in Baidu News include more than 500 authoritative websites, which ensures the comprehensiveness of the information we obtain. Finally, compared with Baidu Search, Baidu News filters more invalid information such as advertising information, thus improving our information processing efficiency.

2 We choose to conduct sentiment analysis on news headlines because investors will not read the contents of the coverage in detail considering their limited time and energy, but rather obtain information through reading news headlines only.
